# A Case of Sick Sinus Syndrome With Prolonged Asystole Masquerading as a Transient Ischemic Attack

**DOI:** 10.7759/cureus.35465

**Published:** 2023-02-25

**Authors:** Azeem Rathore, Nidhi Gupta, Ele Wu, Prakash Suryanarayana, John N Catanzaro

**Affiliations:** 1 Internal Medicine, University of Florida College of Medicine – Jacksonville, Jacksonville, USA; 2 Cardiology/Electrophysiology, University of Florida College of Medicine – Jacksonville, Jacksonville, USA

**Keywords:** cardiac pacemaker, cardiac telemetry, transient ischemic attack, asystole, sick sinus syndrome

## Abstract

Sick sinus syndrome (SSS) is a term used to describe dysfunction of the sinoatrial (SA) node that can lead to various cardiac arrhythmias that predominately manifest in the elderly. Commonly implicated arrhythmias vary from inappropriate bradycardia, tachycardia, sinus pauses, and rarely sinus arrest. Despite being a common reason for permanent pacemaker implantation, little is known regarding the incidence of SSS and there is even less reporting on SSS complicated by prolonged asystole. We present a case highlighting an infrequently observed manifestation of SSS with recurrent, prolonged ventricular asystolic episodes that were causing previously unexplained episodes of confusion and agonal breathing. Our patient was a 75-year-old male with a past medical history of hypertension, dyslipidemia, and prior transient ischemic attacks (TIAs) that presented after an acute mental status change. The initial leading differential diagnosis was believed to be a TIA and he was admitted to neurology service for further evaluation. The patient had recurring episodes of confusion associated with agonal breathing that upon closer review of the cardiac telemetry revealed sinus bradycardia to the 40s interrupted by several prolonged episodes of asystole, the longest lasting 20 seconds. Due to his symptoms and to avoid potential deterioration resulting in hemodynamic instability, the electrophysiology service urgently placed a temporary transvenous pacemaker and then later implanted a leadless pacemaker. On outpatient follow-up, he no longer had episodes of confusion, and no further asystolic episodes were noted on his device check.

## Introduction

Electrical impulses in the heart are initiated by the sinoatrial (SA) node that transmits to the atrioventricular (AV) node within the right atrium followed by a signal that travels through the bundle of His, down the bundle branches, and through the Purkinje fibers resulting in myocardial contraction and blood distribution to the rest of the body. Any abnormality in the electrical conduction system including the SA node, also known as the cardiac pacemaker, can lead to an arrhythmia [1]. One relatively uncommon disorder of the SA node is sick sinus syndrome (SSS) which can be characterized by a spectrum of abnormal rhythms, ranging from sinus bradycardia, sinus pause, SA exit block, paroxysmal atrial tachycardia, atrial fibrillation with slow ventricular rate, or tachycardia-bradycardia arrhythmias [1,2].

In SSS, transient cessation of impulses from the SA node can commonly lead to sinus pauses usually lasting less than three seconds. The “latent pacemakers” present in other portions of the conduction system take over the SA node and generates an escape rhythm; however, in the absence of sinus pauses, this rhythm can last longer than three seconds, which is now referred to as asystole [3]. Hypoperfusion of end organs during these episodes of asystole can manifest with varying clinical presentations ranging from lightheadedness, syncope, angina, palpitations, confusion, and fatigue among others [2]. Indeed, asystole of a very long duration, specifically upwards of 20 seconds, has rarely been reported in patients diagnosed or suspected of SSS. Here, we present a case of a 76-year-old male with a presentation of SSS with prolonged asystole masquerading as a transient ischemic attack (TIA).

## Case presentation

**Figure 1:**
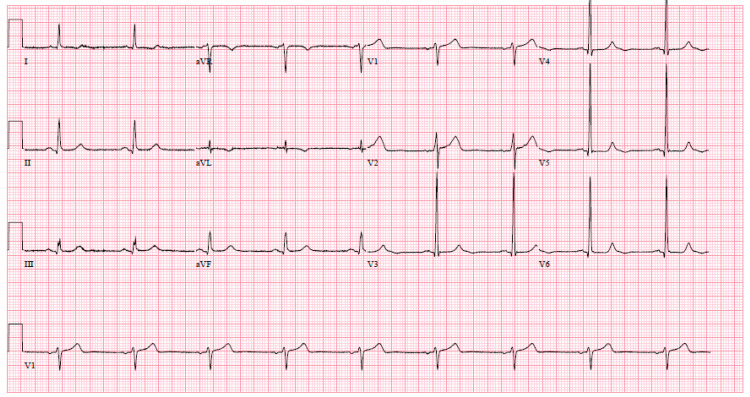
Baseline ECG showing sinus bradycardia (54 bpm) with left ventricular hypertrophy (Sokolow-Lyon criteria) without any signs of acute ischemic disease.

A 75-year-old male with a past medical history of hypertension, dyslipidemia, previous TIAs, and a former smoker with a 30-pack-year history presented to the Emergency Department with sudden aphasia and acute change mentation. According to the wife, she was awakened in the middle of the night having found her husband with agonal breathing, stuporous, and unresponsive to her verbal commands. After what was only a few minutes, he became more responsive to her questioning but with transient word-finding difficulty. Upon initial presentation, the patient was afebrile with a blood pressure of 159/91 mmHg, heart rate of 58 bpm, and oxygen saturation of 97% on room air. Neurology was consulted and upon their assessment, the patient had a National Institutes of Health Stroke Scale (NIHSS) of 2 for mild-moderate expressive aphasia and oriented only to self. Of note, the wife stated his last known normal mentation was right before going to sleep and she also added he has had several similar episodes within the past few years as well. A baseline electrocardiogram (ECG) showed sinus bradycardia without any signs of ischemia (Figure 1). Initial laboratory data included normal hematological and metabolic profiles. A non-contrast computed tomography of the head did not show any signs of acute intracranial pathology. The patient was subsequently admitted to the neurology service for further work-up of a suspected TIA and he returned to his baseline mentation within hours. Follow-up neuroimaging did not elucidate any cerebrovascular process nor were any occlusions or flow-limiting stenoses of the carotid arteries found that could have contributed to cerebral hypoperfusion.

**Figure 2:**
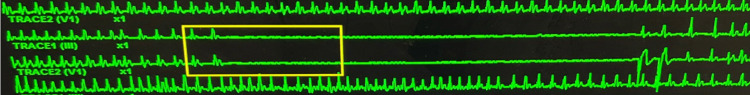
Cardiac telemetry showing a 20-second asystole episode occurring during admission

Overnight an additional episode of transient confusion occurred characterized by agonal breathing that overlapped with a sinus pause on cardiac monitoring. Electrophysiology was consulted and upon review of telemetry, the patient had a total of three significant sinus pauses; the longest lasting upwards to 20 seconds in the setting of sinus bradycardia (Figure 2). A review of the patient's home medications was unrevealing for any that could have precipitated sinus node dysfunction, such as beta-blockers or calcium channel blockers. Electrolytes and thyroid function tests were also normal. Subsequently, the patient was transferred to the cardiac care unit for emergent placement of a transvenous pacemaker for suspected SSS and a leadless permanent pacemaker (Medtronic Micra™, Medtronic plc, Dublin, Ireland) was implanted a few days later.

A follow-up transthoracic echocardiogram noted left ventricular dysfunction with an ejection fraction of 40-45% (Video 1). In order to assess for coronary artery disease burden, a coronary angiogram was done revealing atherosclerotic disease to both mid-left anterior descending and left circumferential coronary arteries that were treated with coronary stents. The patient was discharged on both dual antiplatelet therapy and guideline-directed medical therapy with strict outpatient monitoring. The patient had a one-month follow-up visit with electrophysiology in which he did not report any further episodes of confusion and no additional asystolic episodes had occurred via device check.

**Video 1:** Transthoracic echocardiogram, four-apical view chamber, depicting left ventricular dysfunction with an ejection fraction of 40-45%

## Discussion

SSS affects men and women equally, and while the syndrome can occur at any age it usually manifests in the elderly population as aging tends to lower SA node function. Some studies have shown that in people over the age of 65 with cardiac disease, one out of 600 were diagnosed with SSS with an overall prevalence of about 0.3% in general [1,4]. Patients with SSS are usually asymptomatic early during the disease process; however, as the disease progresses, they may develop symptoms due to cerebral hypoperfusion that can manifest as presyncope or syncope [2]. Indeed, SSS has been one of the leading causes of pacemaker implantation in the United States [2]. Still, early detection of SSS remains difficult due to its paroxysmal nature and asymptomatic dormant state thereby likely leading to the actual incidence of clinically significant disease being much higher than what is reported in the literature.

The etiology of SSS can be intrinsic or extrinsic to the SA node. Intrinsic causes include degenerative fibrosis, infiltrative diseases, connective tissue diseases, ion channel dysfunction, and idiopathic remodeling of the SA node. Additionally, chronic atherosclerosis of the sinus nodal artery (which originates in 65% of individuals from the right coronary artery and the remaining from the left circumflex artery), may also contribute to chronic SSS. For our patient, ischemic heart disease and elderly age were the main risk factors for developing SSS. In fact, after the prolonged episode of 20-second asystole, the patient recovered with a left bundle branch pattern, which itself can be the result of an aging or fibrotic conduction system or chronic ischemic heart disease [2,5]. Further, while there was some suspicion that the patient was experiencing a pause-dependent paroxysmal AV block, defined as the sudden and repetitive block of atrial impulses to the ventricles, it was unlikely given that the p-p interval could not be adequately measured out. As for extrinsic factors, they can mimic or exacerbate SSS and include autonomic dysfunction, carotid sinus hypersensitivity, increased vagal tone, metabolic derangements, hypothermia, hypothyroidism, hypoxia, obstructive sleep apnea, and medications, including antiarrhythmics, beta-blockers, and calcium channel blockers among others [2]. An extensive clinical evaluation and work-up ruled out any extrinsic causes for our patient.

To diagnose SSS, the patient is required to have both clinical symptoms of hypoperfusion and ECG abnormalities, especially bradycardia, with or without accompanying tachycardia [1,5]. The typical electrocardiographic features for diagnosis of SSS include sinus bradycardia, SA pause of three seconds or more, SA exit block, or sinus arrest [1,5]. Our patient met the clinical and ECG criteria for SSS. In the context of the current literature, there is sparse reporting on patients with SSS characterized by prolonged asystole. In one case, an 80-year-old Saudi Arabian patient with SSS was reported to have a documented asystole of 18 seconds with a history of atrial fibrillation [6]. In a coronavirus disease 209 (COVID-19) patient, authors noted multiple, prolonged asystole episodes lasting longer than 20 seconds without an escape rhythm [7].

The definitive treatment for SSS after correcting for reversible causes is a permanent pacemaker, which is recommended for symptomatic patients not in order to improve survival but rather to improve quality of life. Dual chamber pacing is the preferred method in these patients as they have an increased propensity to develop AV block [1,2]. Our patient was also managed with a permanent dual chamber pacemaker and has remained asymptomatic since placement and on subsequent outpatient follow-up visits.

## Conclusions

This case highlights how SSS can be a diagnostically elusive presentation for clinicians, especially when other etiologies are being investigated. Indeed, clinicians should consider wearable or implantable cardiac monitoring in the evaluation of unexplained, recurrent TIAs as part of the workup and management of such patients. If it was not for the pairing of the cardiac telemetry with the recurrence of his symptoms during his hospital stay, the patient would likely have been discharged with a repeat diagnosis of TIA without further intervention. Ultimately, careful evaluation of cardiac telemetry and recognition of signs and symptoms of SSS helped our patient receive prompt appropriate management and avoid a likely fatal outcome.
